# Supplementary figures and images for: Stem-loop structure preference for site-specific RNA editing by APOBEC3A and APOBEC3G
Source: PeerJ. 2017 Dec 6;5:e4136. doi: 10.7717/peerj.4136 (PMC5723131; doi:10.7717/peerj.4136)

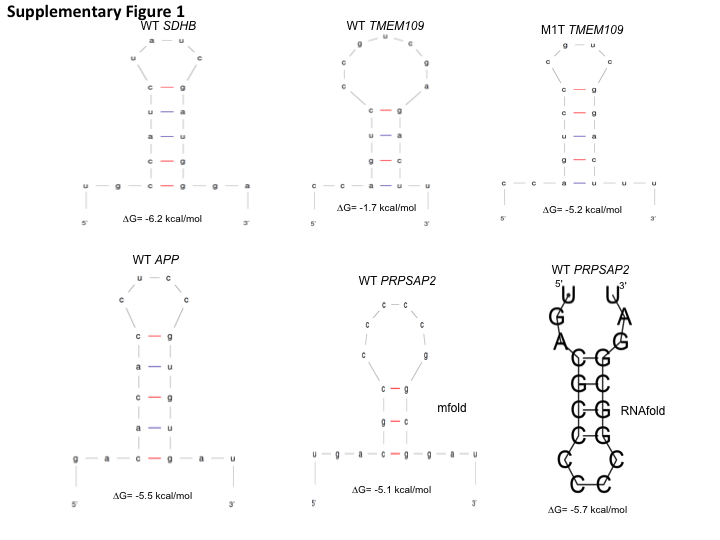

Supplement: Figure S1 — The predicted ΔG values are below each RNA structure. [file peerj-05-4136-s003.png]
